# Supplementary material for: Hemocyanin facilitates lignocellulose digestion by wood-boring marine crustaceans
Source: Nat Commun. 2018 Dec 3;9:5125. doi: 10.1038/s41467-018-07575-2 (PMC6277391; doi:10.1038/s41467-018-07575-2)
Supplement: Supplementary file 1 — Supplementary Information [file 41467_2018_7575_MOESM1_ESM.pdf]

Supplementary Information

Hemocyanin facilitates lignocellulose digestion by wood-boring marine crustaceans

Katrin Besser *et al.* 2018

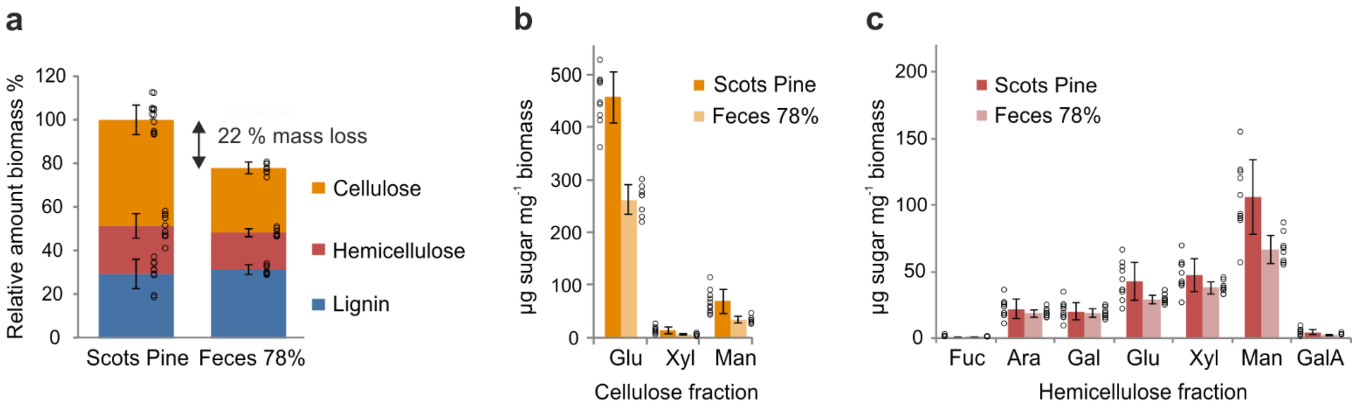

Supplementary Figure 1. Biopolymer composition of Scots pine sapwood before and after digestion. **a** Relative amounts of biomass fractions from wood (Scots Pine, N=9) and fecal pellets (Feces 78 %, normalized to mass loss during digestion, N=8). Fractions comprise acetyl bromide-soluble lignin (blue), TFA-soluble hemicellulose (red) and sulfuric acid-soluble cellulose (orange). **b** Monosaccharide composition (absolute amount) of cellulose fraction from wood before (Scots Pine, N=10; dark orange) and after digestion (Feces 78 %, normalized to mass loss during digestion, N=8; light orange) analyzed by sequential H_2SO_4 hydrolysis following the TFA hydrolysis in (**c**). **c** Monosaccharide composition (absolute amount) of hemicellulose fraction from wood before (Scots Pine, N=10; dark red) and after digestion (Feces 78 %, normalized to mass loss during digestion, N=10; light red) analyzed by TFA hydrolysis. Circles represent sample values and bars sample mean ± SD. Fuc, fucose; Ara, arabinose; Gal, galactose; Glu, glucose; Xyl, xylose; Man, mannose; GalA, galacturonic acid.

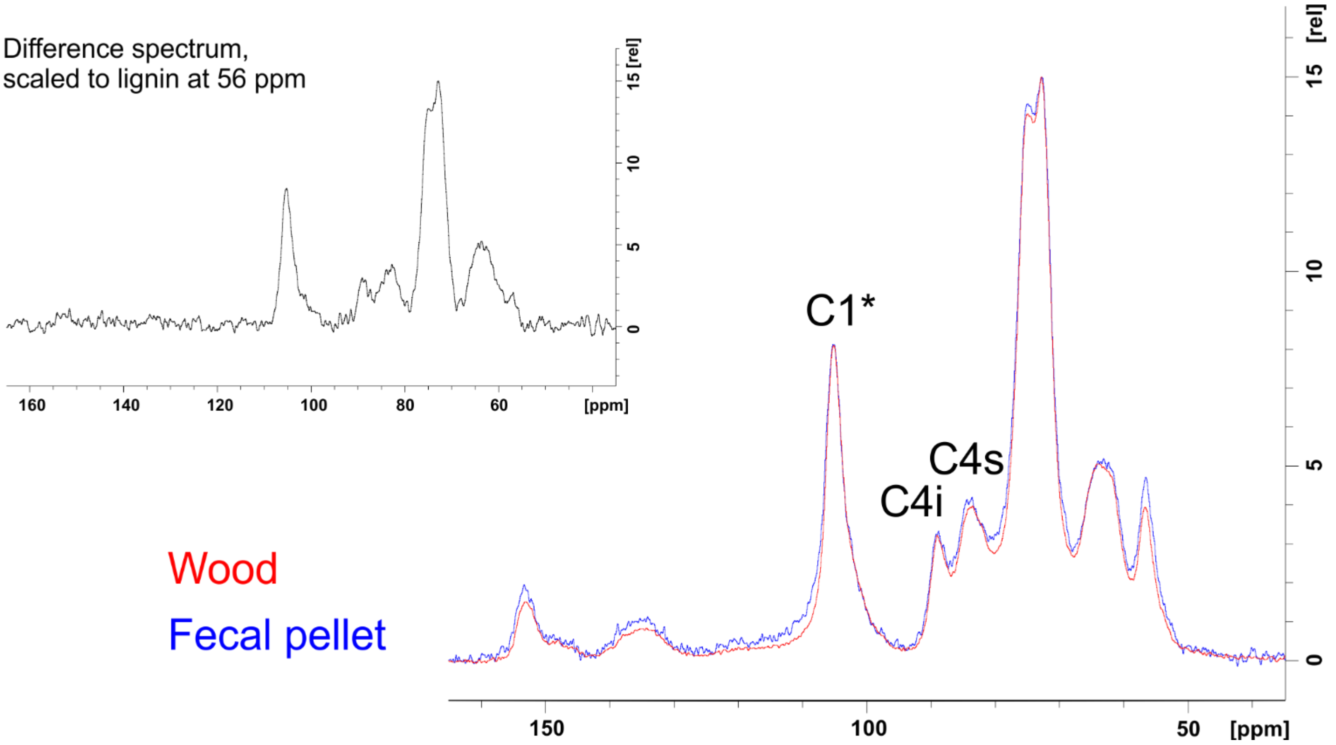

Supplementary Figure 2. ssNMR spectra of willow wood before and after digestion. Spectra of willow wood before (red) and after digestion (blue, fecal pellets) scaled to C1 of cellulose/hemicellulose (*, 105.2 ppm). Relative peak heights of C4 of ordered/internal (i, 89 ppm) and disordered/surface (s, 84 ppm) cellulose remain relatively unchanged in fecal pellets compared to wood [1]. ***Inset***: Difference spectrum of willow wood and fecal pellet spectra scaled to lignin peak height at 56 ppm (aryl methoxyl carbons of lignin). The spectrum resembles most closely a spectrum of cellulose, indicating that mostly cellulose is removed from wood, whereas lignin and hemicellulose accumulate in fecal pellets.

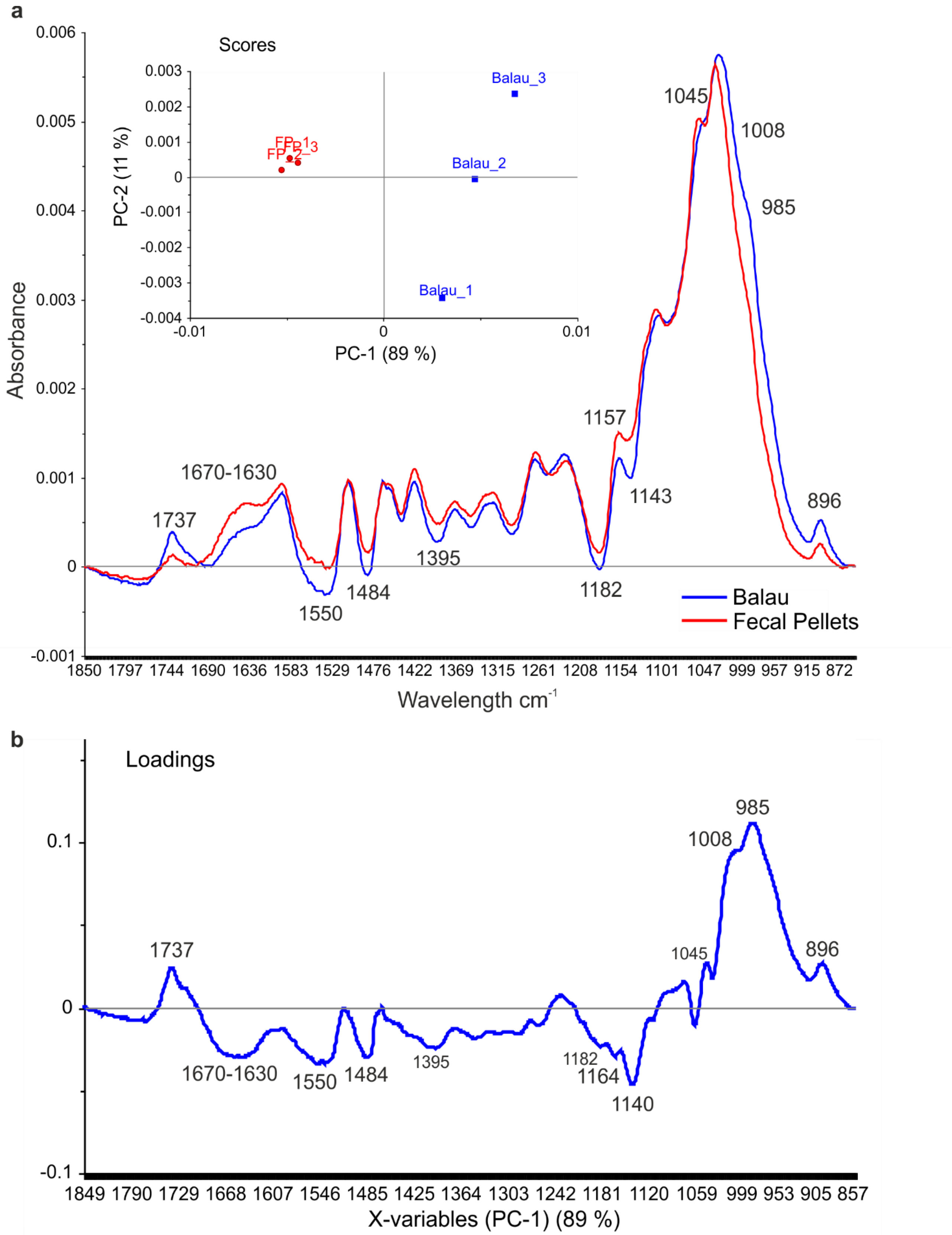

Supplementary Figure 3. ATR-FTIR spectroscopy of wood before and after digestion. a Averaged absorbance spectra of balau wood and fecal pellets (FP) derived thereof (N=3). ***Inset*****:** Scores distribution of PCA with sample clustering on PC-1. **b** Loadings plot indicating wavenumber contributions to PC-1 accounting for 89 % of variation between wood and fecal pellets.

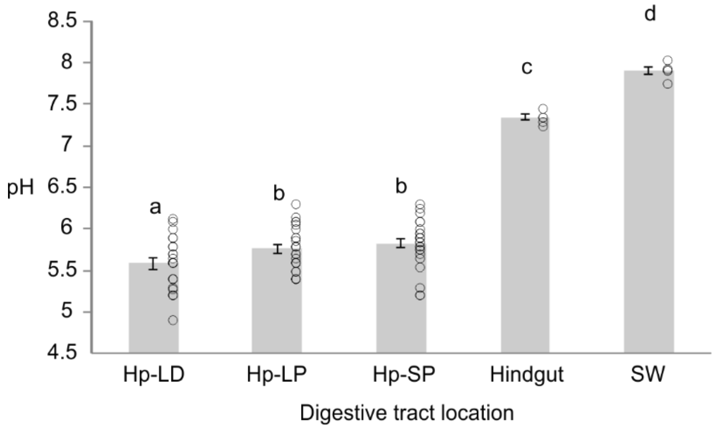

Supplementary Figure 4. Variation of pH in the digestive tract of *Limnoria quadripunctata*. Luminal fluid pH of distal and proximal regions of large hepatopancreas lobes (Hp-LD and Hp-LP, respectively), of proximal region of small lobes (Hp-SP), and of hindgut; pH of surrounding seawater/agarose gel medium supporting the hindgut (SW). Circles represent sample values and bars sample mean ± SE, with N=24 for measurements from left and right lobes of 12 animals and with N=5 for measurements of hindgut from 5 animals and SW adjacent to these. Letters indicate statistically distinct mean values. Data from all five regions analysed by Welch's one-way ANOVA with Games-Howell comparison, $F(4,18.28) = 316.38$, $p < 0.0005$. Differences between hepatopancreas regions were analysed by GLM ANOVA with Tukey pairwise comparisons (model factors: proximal v distal, $F(2,22) = 10.34$, $p = 0.001$; left v right, $F(1,22) = 0.04$, $p = 0.837$; between animals, $F(11,22) = 8.10$, $p < 0.0005$). There were no significant factor interactions.

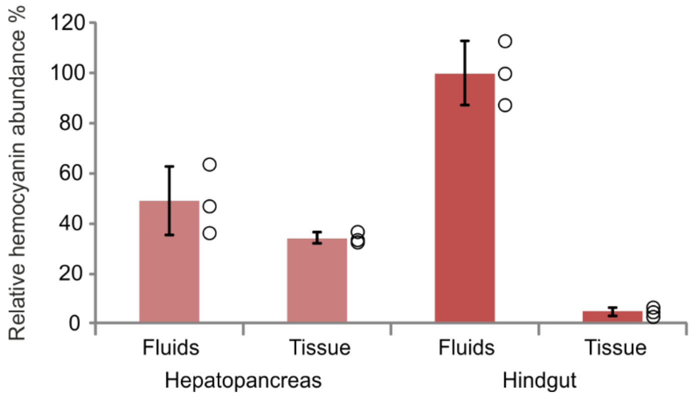

Supplementary Figure 5. Hemocyanin accumulation in the *Limnoria* digestive system. Relative hemocyanin abundance in hepatopancreas and hindgut, separated into tissue (including solid content) and fluids each, by comparison of relative molar percentage of hemocyanins. Circles represent sample values and bars sample mean ± SD, N=3 (N consisting of material from 100 animals each), replicates were normalized intra-sample on the expectation of equal total protein amount between replicates when summed across all organs.

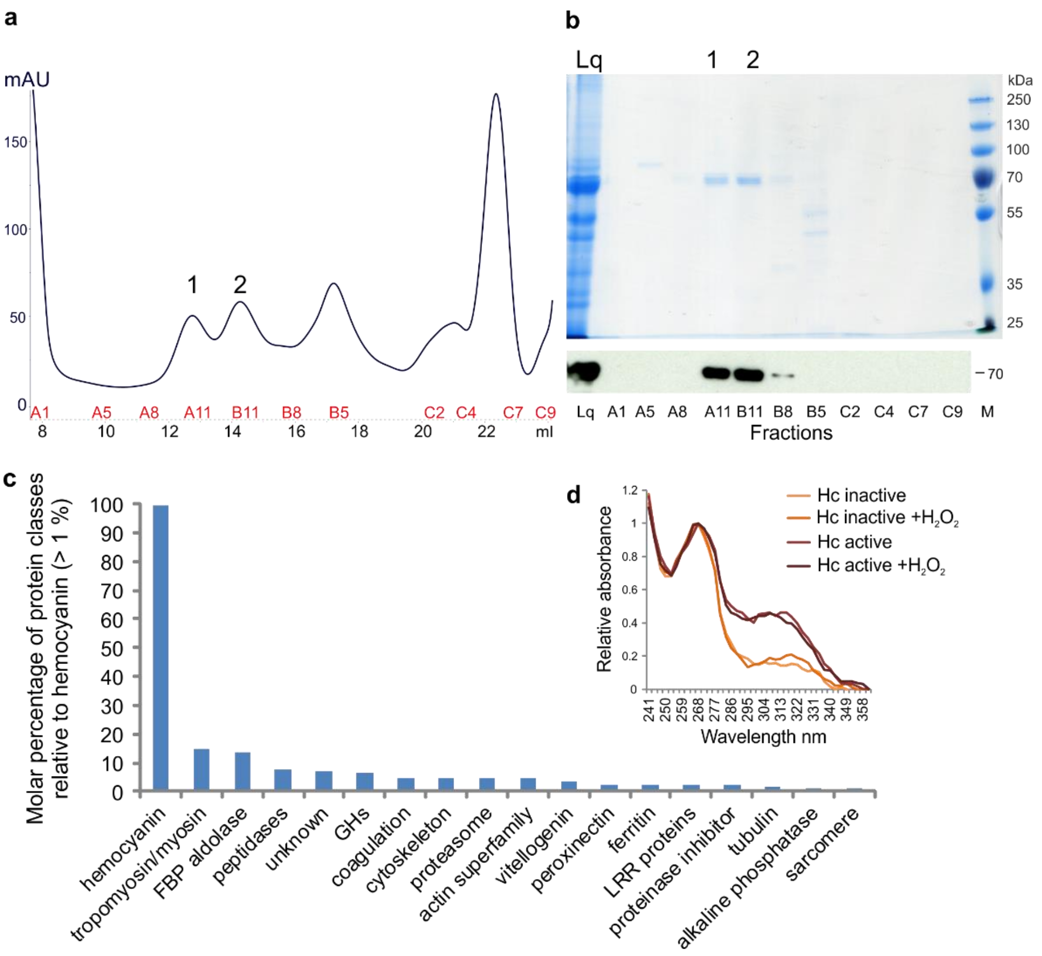

Supplementary Figure 6. Purification of native *Limnoria* hemocyanin. a UV-trace of whole body protein gel filtration (fractionated in A 1-12, B12-1, C1-9). Fractions of peaks 1 and 2 contain hemocyanin (see b). **b** Anti-hemocyanin Western analysis of fractions collected during gel filtration as indicated after separation on SDS-PAGE gels compared to whole body extract (Lq). Top panel shows Coomassie blue-stained gel of same fractions and molecular size marker in kDa (M), bottom panel shows fluorescence signal after detection with anti-hemocyanin antibodies. **c** LC-MS/MS analysis of purified and concentrated hemocyanin from fractions of peaks 1 and 2 of gel filtration in (a). EmPAI-derived molar percentages of identified protein classes relative to hemocyanin (> 1 %) in native *Limnoria* hemocyanin extract (FBP=Fructose-1,6-bisphosphate; GHs=glycosyl hydrolases; LRR=leucine-rich repeat). **d** Note on peroxinectin, which is an arthropod opsonic peroxidase that plays a role in invertebrate immunity mechanisms in the hemolymph: This enzyme was not detected in the gut fluid proteome (PXD009486; MSV000082271) and no peroxidase activity had been detected in the hemocyanin extract during incubation with 1 mM pyrogallol when supplemented with 1 mM hydrogen peroxide without activation of hemocyanin (by addition of 2.75 mM SDS). UV-Vis spectra represent averages from duplicate reactions each in 0.1 M $NaPO_4$ pH 6.8 for 20 min at room temperature with 3.3 µM hemocyanin. Absorbance maximum of pyrogallol at 267 nm and of oxidation product purpurogallin at ~320 nm.

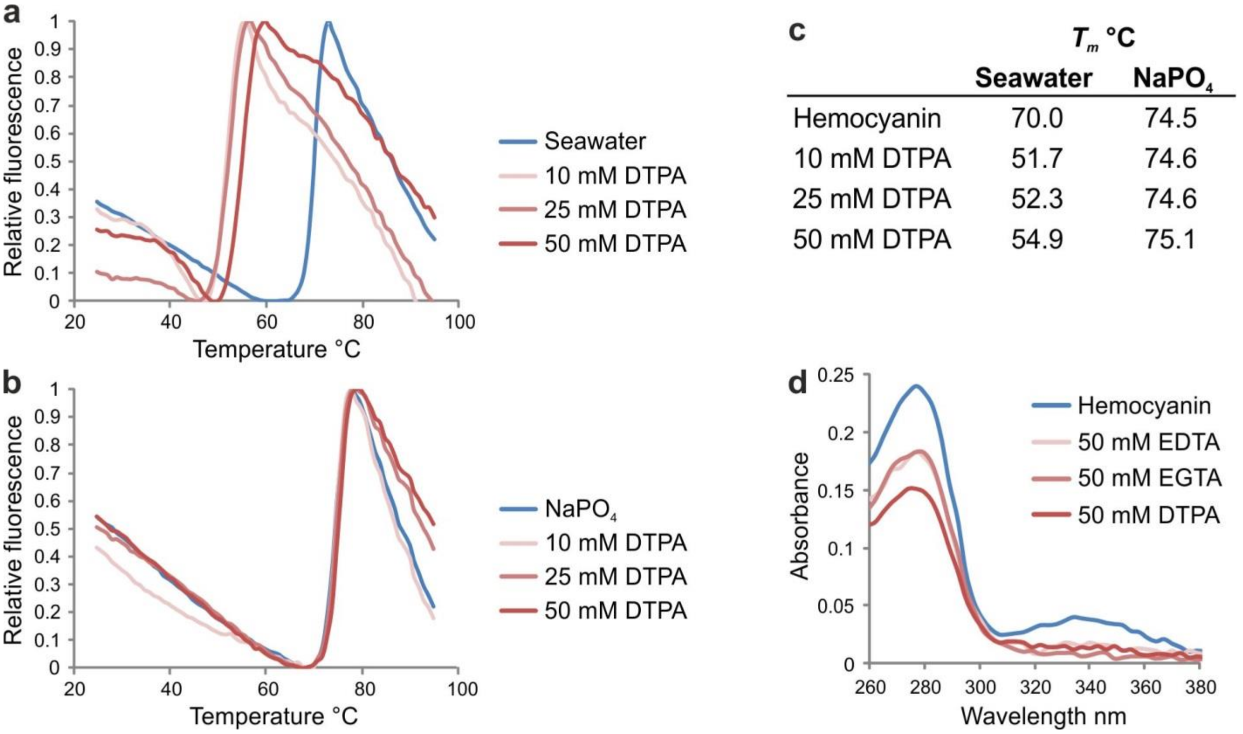

Supplementary Figure 7. Thermal shift assay and UV-Visible absorbance of *Limnoria* hemocyanin. Temperature-dependent SYPRO™ Orange fluorescence profiles of hemocyanin (blue) in seawater (**a**) or in 0.05 M $NaPO_4$ buffer pH7 (**b**), and after chelator addition of 10 to 50 mM DTPA (red shades; **a, b**). **c** Melting temperatures (T_m) in seawater and buffer and with various DTPA concentrations: T_m stable in buffer (~74.5 °C), but shifts to 70 °C in seawater and further to below 55 °C in the presence of chelator. **d** UV-visible absorbance spectra of hemocyanin in seawater (blue) and after addition of various chelators (50 mM each of EDTA, EGTA, DTPA; red shades), loss of absorbance at ~340 nm indicates removal of oxygen from the active site [2, 3].

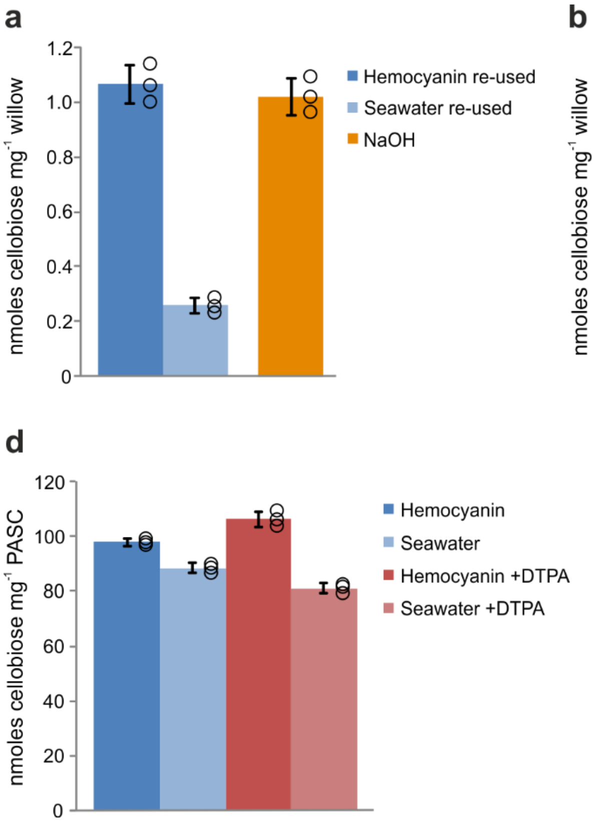

Supplementary Figure 8. Comparative digestibility assays of hemocyanin-treated wood and cellulose. Digestibility of willow, Scots pine or cellulose (PASC) measured as release of cellobiose (nmoles) per unit biomass (mg) in saccharification reactions upon biomass pretreatment. **a** Willow pretreated for 10 min at room temperature with 14 µM hemocyanin or seawater that had both been used in a previous pretreatment reaction on willow (blue bars), or pretreated for 45 minutes at 90 °C with 0.5 N NaOH (orange bar), followed by saccharification with *Hj*CBH I. **b** Comparing saccharification of willow pretreated with 14 µM hemocyanin for 15 min at room temperature using the fungal *Hj*CBH I versus the limnoriid *Lq*Cel7B **c** Scots pine sapwood pretreated for 10 min at room temperature with 14 µM hemocyanin or seawater, followed by saccharification with *Lq*Cel7B. **d** PASC pretreated for 10 min at room temperature with 14 µM hemocyanin or seawater with (+) and without 50 mM DTPA, followed by saccharification with *Hj*CBH I. Hydrolysates were analyzed by HPAEC against cello-oligo standards, and values (apart from Scots pine samples in **c**) normalized to reactions without CBH following pretreatments to account for any remaining cellulase activity derived from the hemocyanin preparation. Circles represent sample values and bars sample mean ± SD, N=3 (N=2 for fungal sample in **b**).

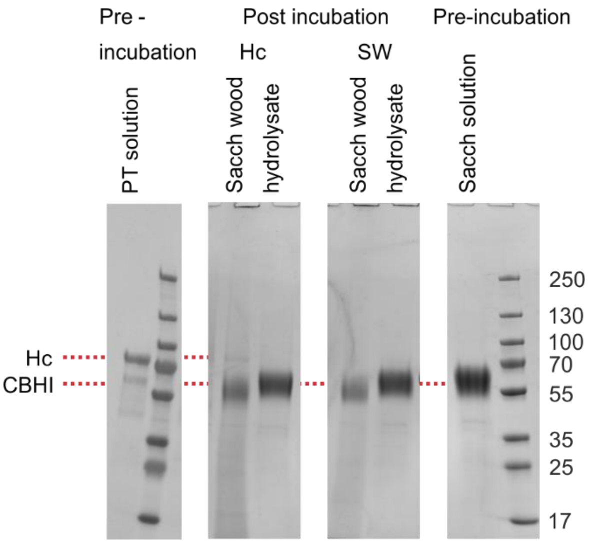

Supplementary Figure 9. Cellulase binding to hemocyanin-pretreated biomass. Analysis of protein content in the biomass (Sacch wood) and hydrolysates after saccharification of hemocyanin or seawater pretreated willow wood powder, assessed by Coomassie-stained SDS-PAGE gels in comparison to the pretreatment (PT) and saccharification (Sacch) solutions prior to incubation. Pretreatment reactions of willow with hemocyanin (Hc) or seawater (SW) for 10 minutes, followed by saccharification with CBH I from *Hypocrea jecorina* (*Hj*CBH I). Comparable volumes loaded onto gel; numbers on the right indicate the molecular sizes of the marker in kDa.

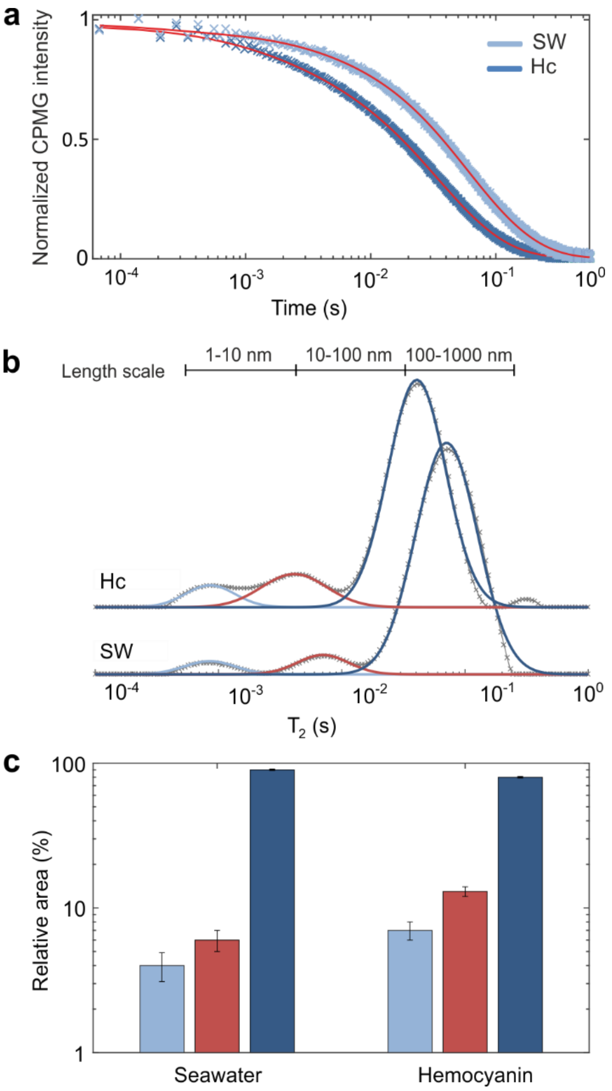

Supplementary Figure 10. Porosity measurements of hemocyanin-pretreated lignocellulose. a Normalized echo intensity obtained in a Carr-Purcell-Meiboom-Gill NMR experiment (CPMG decays) for N,N-Dimethylacetamide (DMAc) in willow wood after hemocyanin (Hc, dark blue) or seawater (SW, light blue) incubation. The faster decay rate of the signal from hemocyanin-treated samples, i.e. lower intensities at longer decay times, may indicate a reduction in the pore sizes or changes in the surface affinity to DMAc. **b** T_2 relaxation time distribution profiles obtained from the Inverse Laplace Transform (ILT) of the CPMG decays for both samples [4, 5]. The fit to the experimental data using these distributions are shown as continuous red line in (**a**). The log-Gaussian shaped curves represent three T_2 regions corresponding to relaxation times of DMAc inside interstitial spaces (pores) with estimated length scales in the order of 1 to 10 nm (light blue), 10 to 100 nm (red) and 100 to 1000 nm (dark blue) [6]; the small peak at long T_2 in hemocyanin (Hc) treated wood is an artefact of the ILT. These pore populations are interpreted as interstitial spaces in the amorphous fraction of cellulose (surface of cellulose fibrils), voids in the inter-microfibril spaces, and micrometer lumens of the wood, respectively [7]. **c** Relative peak areas of T_2 profiles in (**b**) representing the amount of DMAc molecules inside each pore population in hemocyanin- and seawater-treated willow wood; estimated length scales of pore populations: in the order of 1 to 10 nm (light blue), 10 to 100 nm (red) and 100 to 1000 nm (dark blue); bars represent sample mean ± SD of the fitted parameters of the T_2 distribution deconvolutions from 32 scans (technical replicates).

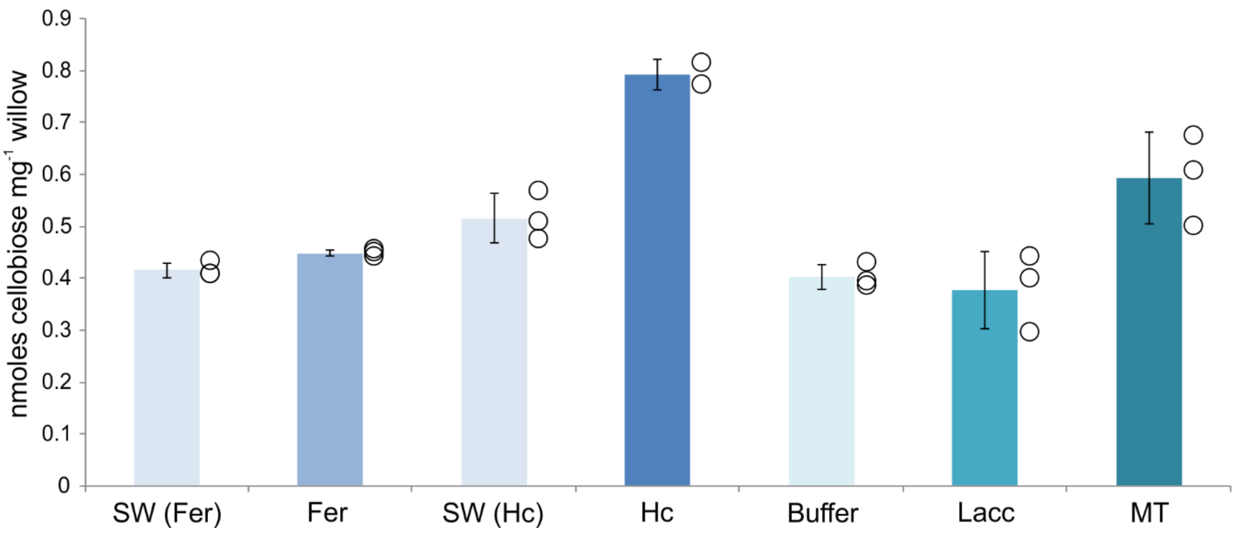

Supplementary Figure 11. Pretreatment efficiency of ferritin, laccase and mushroom tyrosinase. Digestibility of willow measured as release of cellobiose (nmoles) per unit biomass (mg) in saccharification reactions upon biomass pretreatment. Pretreatment of willow for 15 minutes at room temperature with 1.9 µM horse spleen ferritin (Fer) or seawater (SW [Fer]), or with 14 µM *Trametes versicolor* laccase (Lacc) or *Agaricus bisporus* mushroom tyrosinase (MT) each or buffer controls, followed by saccharification with *Hj*CBH I and compared to pretreatment with 14 µM hemocyanin (Hc) or seawater (SW [Hc]) as of Fig. 5 a. Hydrolysates were analyzed by HPAEC against cello-oligo standards, and values normalized to reactions without CBH following pretreatments. Circles represent sample values and bars sample mean ± SD, N=3 (N=2 for Hc).

Figure 3 c

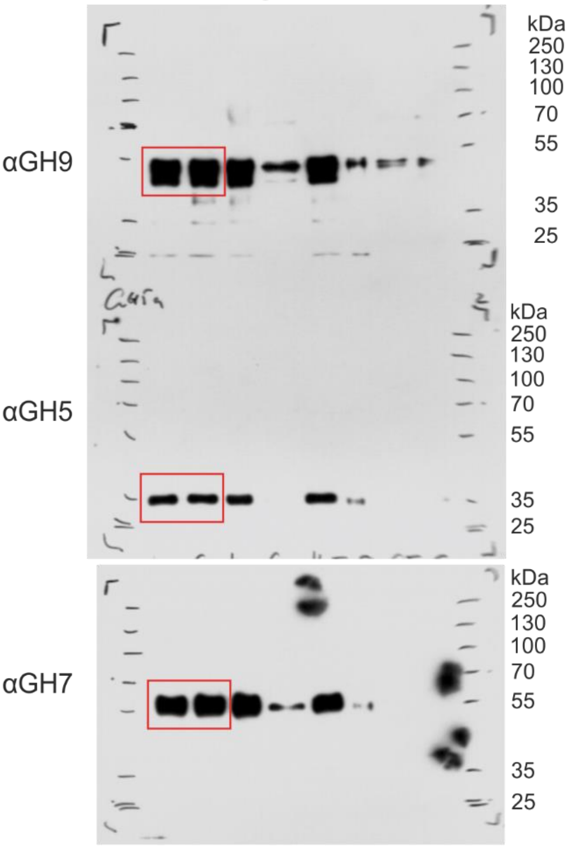

Figure 3 d αHemocyanin

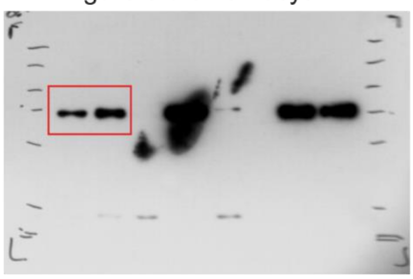

Figure 3 e

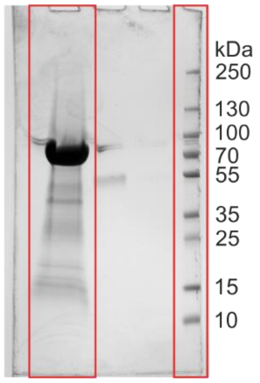

Supplementary Figure 6 b αHemocyanin

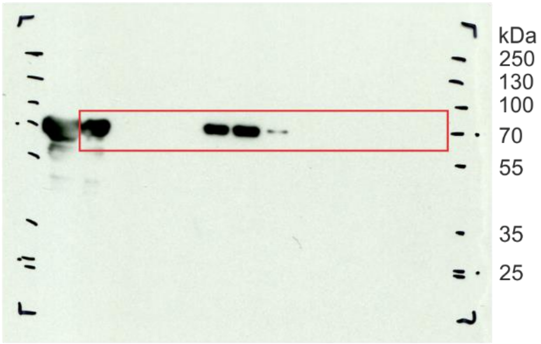

Supplementary Figure 9

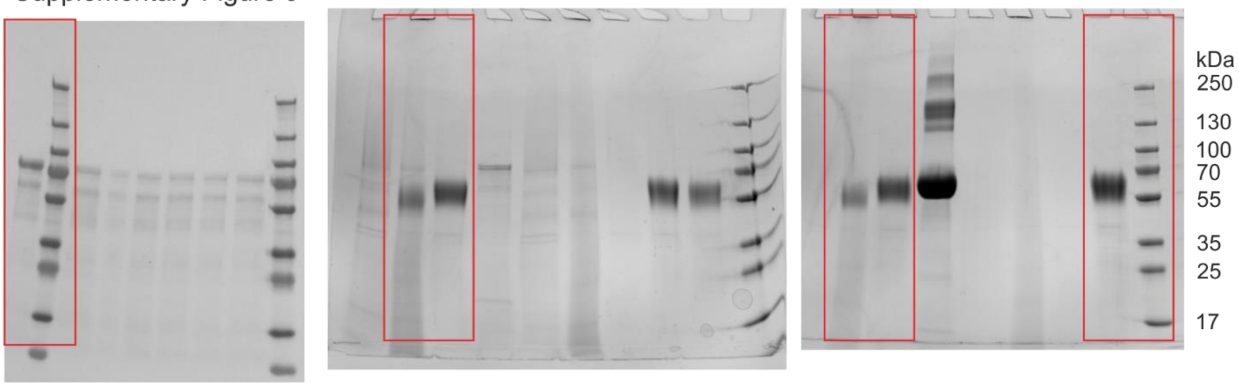

Supplementary Figure 12. Uncropped Western blots and Coomassie-stained SDS PAGE gels.

Uncropped images of all blots and gels; red boxes indicate cropping used in respective figures.

Supplementary Table 1. Mass loss during digestion of wood by *Limnoria quadripunctata*

	Willow				Scots Pine			
**N**	**Mass removed[a]**	**Fecal mass produced**	**Mass consumed[b]**	**% Mass loss[c]**	**Mass removed[a]**	**Fecal mass produced**	**Mass consumed[b]**	**% Mass loss[c]**
1	0.013	0.0101	0.0029	22.4	0.020	0.0156	0.0048	23.6
2	0.017	0.0122	0.0044	26.7	0.024	0.0189	0.0055	22.6
3	0.016	0.0130	0.0029	18.3	0.020	0.0160	0.0044	21.8
4	0.018	0.0143	0.0036	20.0	0.025	0.0206	0.0048	19.0
5	0.015	0.0110	0.0036	24.6	0.023	0.0176	0.0050	21.9
6	0.014	0.0119	0.0023	16.4	0.025	0.0195	0.0052	21.0
7	0.015	0.0109	0.0038	25.7	0.022	0.0171	0.0049	22.3
8	0.016	0.0115	0.0040	25.8	0.022	0.0158	0.0059	27.3
9	0.020	0.0163	0.0033	16.8	0.021	0.0165	0.0045	21.5
10	0.016	0.0128	0.0035	21.4	0.021	0.0166	0.0043	20.6
**x**				**21.8 ± 3.8**				**22.2 ± 2.2**

Animals were fed on wood sticks (willow or Scots pine sapwood) and fecal pellets collected. [a] Mass removed = wood weight before - after feeding; [b] Mass consumed = mass removed[a] - fecal mass produced; [c] Percent mass loss was calculated as ratio of mass consumed[b] to mass removed[a] in percent. x = sample mean ± SD, N=10.

Supplementary Table 2. ATR-FTIR spectral assignments of wood and fecal pellet absorbance with highest contribution to PC-1

***Limnoria* fecal pellets compared to wood**			**Other studies**	
**Wavenumber cm^{-1}**	**Absorbance in FP**	**Indicated modification**	**Wavenumber cm^{-1}**	**Assignment**
**1737**	decreased	**reduced acetylation** [8] **and 4-*O*-methyl-glucuronylation** [9] **of xylan**	1740-1710	C=O stretching (unconjugated ketone, ester or carboxylic groups) [8-10]
**1670-1630**	increased	**lignin accumulation and oxidation**	1675-1660<br>1655	C=O stretching (conjugated to aromatic ring) [10, 11]
			1650-1600	C=O stretching (conjugated to aromatic ring); aromatic ring vibrations [8]
			1640	quinononid structure (oxidation of aromatic ring into quinononid) [12]
**1550**	increased	**lignin oxidation**	1550	oxidised lignin structures or protein [8]
**1484**	increased	**?**		
**1395**	increased	?		
**1182**	increased	formation of polylactic acid?	1182	C-O stretching in CH-O (of polylactic acid) [13]
**1160**	increased	**hemicellulose/lignin accumulation**	1157-1161	C-O-C antisymmetric stretching (cellulose, hemicellulose) [14, 15]
			1160	C-H stretching in benzene rings [11]
**1140**	increased	**lignin accumulation**	1140	C-H in plane deformation in guaiacyl rings, plus secondary alcohols plus C=O stretch (soft-/hardwood lignin) [16]
			1140	C-H in plane stretching in guaiacyl rings [11]
**1045**	increased	accumulation of xylan	1047	Branched (1-4)-β-xylans such as glucuronoxylan and arabinoxylan [14]
**1008**	decreased	**less cellulose**	1003	C-O and ring stretching in developing cotton fibers [9]
			998	C-O stretching (cellulose, hemicellulose) [15]
**985**	decreased	**less cellulose**	986	C-O and ring stretching in developing cotton fibers [9]
**896**	decreased	**less cellulose**	897	C-H deformation in cellulose [15]
			896	Character of cellulose P-chains, C-H stretching out of plane of aromatic rings [17]

Supplementary Note 1

Chemical shifts for ^{13}C solid-state NMR spectra of alkali lignin shown in Figure 4 b were assigned based on literature data [18-20].

Supplementary References

1. Park, S., Baker, J.O., Himmel, M.E., Parilla, P.A., Johnson, D.K. Cellulose crystallinity index: measurement techniques and their impact on interpreting cellulase performance. *Biotechnol. Biofuels* **3**, 10 (2010).

2. Decker, H. *et al.* Similar enzyme activation and catalysis in hemocyanins and tyrosinases. *Gene* **398**, 183-191 (2007).

3. Coates, C.J., Nairn, J. Diverse immune functions of hemocyanins. *Dev. Comp. Immunol.* **45**, 43-55 (2014).

4. Borgia, G., Brown, R., Fantazzini, P. Uniform-penalty inversion of multiexponential decay data. *J. Magn. Reson.* **132**, 65-77 (1998).

5. Provencher, S.W. A constrained regularization method for inverting data represented by linear algebraic or integral equations. *Computer Physics Communications* **27**, 213-227 (1982).

6. Zhang, C. *et al.* Hierarchical porous structures in cellulose: NMR relaxometry approach. *Polymer* **98**, 237-243 (2016).

7. Tsuchida, J.E. *et al.* Nuclear magnetic resonance investigation of water accessibility in cellulose of pretreated sugarcane bagasse. *Biotechnol. Biofuels* **7**, 127 (2014).

8. Nuopponen, M.H. *et al.* Characterization of 25 tropical hardwoods with Fourier transform infrared, ultraviolet resonance Raman, and 13C-NMR cross-polarization/magic-angle spinning spectroscopy. *J. Appl. Polym. Sci.* **102**, 810-819 (2006).

9. Abidi, N., Cabrales, L., Haigler, C.H. Changes in the cell wall and cellulose content of developing cotton fibers investigated by FTIR spectroscopy. *Carbohydr. Polym.* **100**, 9-16 (2014).

10. Hoareau, W., Trindade, W.G., Siegmund, B., Castellan, A., Frollini, E. Sugar cane bagasse and curaua lignins oxidized by chlorine dioxide and reacted with furfuryl alcohol: characterization and stability. *Polym. Degradation Stab.* **86**, 567-576 (2004).

11. Ke, J., Singh, D., Yang, X., Chen, S. Thermal characterization of softwood lignin modification by termite Coptotermes formosanus (Shiraki). *Biomass Bioenergy* **35**, 3617-3626 (2011).

12. Sun, R., Tomkinson, J., Wang, S., Zhu, W. Characterization of lignins from wheat straw by alkaline peroxide treatment. *Polym. Degradation Stab.* **67**, 101-109 (2000).

13. Fortunati, E., Puglia, D., Santulli, C., Sarasini, F., Kenny, J. Biodegradation of phormium tenax/poly (lactic acid) composites. *J. Appl. Polym. Sci.* **125**, (2012).

14. Kacurakova, M., Capek, P., Sasinkova, V., Wellner, N., Ebringerova, A. FT-IR study of plant cell wall model compounds: pectic polysaccharides and hemicelluloses. *Carbohydr. Polym.* **43**, 195-203 (2000).

15. Çetinkol, Ö.P. *et al.* Understanding the impact of ionic liquid pretreatment on eucalyptus. *Biofuels* **1**, 33-46 (2010).

16. Casas, A., Oliet, M., Alonso, M., Rodriguez, F. Dissolution of Pinus radiata and Eucalyptus globulus woods in ionic liquids under microwave radiation: lignin regeneration and characterization. *Sep. Purif. Technol.* **97**, 115-122 (2012).

17. Chen, H. *et al.* Qualitative and quantitative analysis of wood samples by Fourier transform infrared spectroscopy and multivariate analysis. *Carbohydr. Polym.* **82**, 772-778 (2010).

18. Bardet, M., Foray, M.F., Tran, Q.K. High-resolution solid-state CPMAS NMR study of archaeological woods. *Anal. Chem.* **74**, 4386-4390 (2002).

19. Landucci, L.L., Ralph, S.A. Assessment of lignin model quality in lignin chemical shift assignments - Substituent and solvent effects. *J. Wood Chem. Technol.* **17**, 361-382 (1997).

20. Rezende, C.A. *et al.* Chemical and morphological characterization of sugarcane bagasse submitted to a delignification process for enhanced enzymatic digestibility. *Biotechnol. Biofuels* **4**, 1-18 (2011).
