# Supplementary material for: Hemocyanin facilitates lignocellulose digestion by wood-boring marine crustaceans
Source: Nat Commun. 2018 Dec 3;9:5125. doi: 10.1038/s41467-018-07575-2 (PMC6277391; doi:10.1038/s41467-018-07575-2)
Supplement: Supplementary file 3 — Reporting Summary [file 41467_2018_7575_MOESM3_ESM.pdf]

nature research

Corresponding author(s): Simon McQueen-Mason, Neil Bruce

Reporting Summary

Nature Research wishes to improve the reproducibility of the work that we publish. This form provides structure for consistency and transparency in reporting. For further information on Nature Research policies, see Authors & Referees and the Editorial Policy Checklist.

Statistical parameters

When statistical analyses are reported, confirm that the following items are present in the relevant location (e.g. figure legend, table legend, main text, or Methods section).

n/a	Confirmed	
☐	☒	The exact sample size (*n*) for each experimental group/condition, given as a discrete number and unit of measurement
☐	☒	An indication of whether measurements were taken from distinct samples or whether the same sample was measured repeatedly
☐	☒	The statistical test(s) used AND whether they are one- or two-sided<br>*Only common tests should be described solely by name; describe more complex techniques in the Methods section.*
☐	☒	A description of all covariates tested
☐	☒	A description of any assumptions or corrections, such as tests of normality and adjustment for multiple comparisons
☐	☒	A full description of the statistics including central tendency (e.g. means) or other basic estimates (e.g. regression coefficient) AND variation (e.g. standard deviation) or associated estimates of uncertainty (e.g. confidence intervals)
☐	☒	For null hypothesis testing, the test statistic (e.g. *F*, *t*, *r*) with confidence intervals, effect sizes, degrees of freedom and *P* value noted<br>*Give P values as exact values whenever suitable.*
☒	☐	For Bayesian analysis, information on the choice of priors and Markov chain Monte Carlo settings
☒	☐	For hierarchical and complex designs, identification of the appropriate level for tests and full reporting of outcomes
☒	☐	Estimates of effect sizes (e.g. Cohen's *d*, Pearson's *r*), indicating how they were calculated
☐	☒	Clearly defined error bars<br>*State explicitly what error bars represent (e.g. SD, SE, CI)*

Our web collection on statistics for biologists may be useful.

Software and code

Policy information about availability of computer code

Data collection	HPAEC: Chromeleon 6.8 Chromatography Data Systems software (Dionex); ssNMR: TopSpin version 3.5 (Bruker); ATR-FTIR: Spectrum version 5.0.1 (Perkin-Elmer); pH profiles: SensorTrace Suite software (Unisense); Proteomics/protein ID: Xcalibur software version 4.0 (Thermo); RT-qPCR: StepOnePlus™ Real-Time PCR System (Life Technologies); Gel filtration: UNICORN 5.31 workstation (GE Healthcare); NMR relaxometry: Bruker minispec software v2.59.
Data analysis	HPAEC: Chromeleon 6.8 Chromatography Data Systems software (Dionex); ssNMR: TopSpin version 3.5 (Bruker), OriginLabOriginPro 9.0.0 SR2; ATR-FTIR: Unscrambler X software version 10.5 (CAMO); pH profiles: Minitab version 17.3.1 (Minitab); Transcriptomics: Trinity software v2.5.1 (Trinity), SAMtools; Proteomics: MSConvert (ProteoWizard 3.0.9974), Mascot Daemon version 2.5.1 (Matrix Science); Protein ID: Bruker flexAnalysis software version 3.3), Mascot version 2.4 (Matrix Science Ltd.), Bruker ProteinScape interface version 2.1; RT-qPCR: StepOnePlus™ Real-Time PCR System (Life Technologies); NMR relaxometry: MATLAB R2015a (MathWorks), ILT MATLAB code from Schlumberger Doll Inc.

For manuscripts utilizing custom algorithms or software that are central to the research but not yet described in published literature, software must be made available to editors/reviewers upon request. We strongly encourage code deposition in a community repository (e.g. GitHub). See the Nature Research guidelines for submitting code & software for further information.

Data

Policy information about availability of data

All manuscripts must include a data availability statement. This statement should provide the following information, where applicable:

- Accession codes, unique identifiers, or web links for publicly available datasets
- A list of figures that have associated raw data
- A description of any restrictions on data availability

All transcriptomic raw sequence data were deposited in the NCBI BioProject database (PRJNA453115 [http://www.ncbi.nlm.nih.gov/bioproject/453115]) and Sequence Read Archive (SRA) (SRP142516 [https://www.ncbi.nlm.nih.gov/sra/SRP142516]). All proteomic data sets, including raw data files, processed peak lists, and database search results are available to download from MassIVE (accessions MSV000082271 [ftp://massive.ucsd.edu/MSV000082271]) and ProteomeXchange (accession PXD009486 [http://proteomecentral.proteomexchange.org/cgi/GetDataset?ID=PXD009486]). All other data are available from the corresponding author upon reasonable request.

Field-specific reporting

Please select the best fit for your research. If you are not sure, read the appropriate sections before making your selection.

☒ Life sciences ☐ Behavioural & social sciences ☐ Ecological, evolutionary & environmental sciences

For a reference copy of the document with all sections, see nature.com/authors/policies/ReportingSummary-flat.pdf

Life sciences study design

All studies must disclose on these points even when the disclosure is negative.

Sample size	sample size was chosen so that meaningful conclusions could be drawn, in relation to method, material and resources required for analysis.
Data exclusions	data was only excluded if there was a technical error in reporting on a sample.
Replication	experimental samples were set up as biological replicates where possible, or experiments were at least repeated once and lead to similar results, or samples represented material that was randomly collected over a time range and derived from animal populations.
Randomization	material samples and healthy appearing animals were allocated randomly into experimental groups
Blinding	no blinding was used; all samples were analyzed in the same manner

Reporting for specific materials, systems and methods

Materials & experimental systems

n/a	Involved in the study	
☒	☐	Unique biological materials
☐	☒	Antibodies
☒	☐	Eukaryotic cell lines
☒	☐	Palaeontology
☐	☒	Animals and other organisms
☒	☐	Human research participants

Methods

n/a	Involved in the study	
☒	☐	ChIP-seq
☒	☐	Flow cytometry
☒	☐	MRI-based neuroimaging

Antibodies

Antibodies used	polyclonal sheep anti-LqGH5A and anti-LqGH7A antibodies (Scottish National Blood Transfusion Service, Penicuik, UK); polyclonal rabbit anti-LqGH9A and anti-hemocyanin antibodies (Covalab S.A.S., France);<br>goat anti-rabbit IgG (H+L) secondary antibody, HRP (Invitrogen, #32460); secondary rabbit anti-sheep IgG antibody, HRP conjugate (Sigma, #AP147P)
Validation	To enrich the sera for antigen-specific antibodies, affinity columns were made using recombinant and purified LqGH5A (GU066826), LqGH7A (FJ940756), LqGH9A (FJ940759) or His-tagged LqHc3 (GU166297). Recombinant protein preparations were dialyzed against coupling buffer (0.1 M $NaHCO_3$, 0.5 M NaCl, pH 8.3) and bound to CNBr-activated Sepharose™ 4 Fast Flow resin

(GE Healthcare Life Sciences) followed by affinity purification of an aliquot of each crude antibody serum according to the resin manufacturer's instructions. Purified antibody fractions were characterized for their affinity to each Lq antigen by dot and Western blot using both recombinant Lq protein and Limnoria quadripunctata whole body extracts. Fractions showing the highest titer and no unspecific binding were selected for Western Blot experiments.

Animals and other organisms

Policy information about studies involving animals; ARRIVE guidelines recommended for reporting animal research

Laboratory animals	The study did not involve laboratory animals.
Wild animals	The study did not involve wild animals.
Field-collected samples	Limnoria quadripunctata Holthuis were collected from a heavily infested piece of balau wood (Shorea sp.) removed from a site in the intertidal zone at Portsmouth, UK. Limnoria tripunctata Menzies were collected from heavily infested unidentified wood removed from the intertidal zone around the Isle of Wight, UK. Both species were used to set up laboratory cultures in seawater tanks, either with continuous flow or in stationary cultures, with aerated and regularly exchanged seawater, at ambient temperatures.
